# Impact of SARS-CoV-2 infection on patients with myasthenia gravis: a retrospective study in a Chinese population

**DOI:** 10.3389/fneur.2024.1482932

**Published:** 2024-12-11

**Authors:** Peng Liu, Mengna Li, Liqing Li, Wenli Jia, Huimin Dong, Guoyan Qi

**Affiliations:** ^1^Center of Treatment of Myasthenia Gravis, People’s Hospital of Shijiazhuang, Shijiazhuang, China; ^2^Hebei Provincial Key Laboratory of Myasthenia Gravis, Shijiazhuang, China; ^3^Hebei Provincial Clinical Research Center for Myasthenia Gravis, Shijiazhuang, China

**Keywords:** SARS-CoV-2 infection, COVID-9, myasthenia gravis, symptoms, severity, autoimmunity

## Abstract

**Background and purpose:**

Myasthenia gravis (MG) is characterized by fluctuating muscle weakness due to immune-mediated damage to acetylcholine receptors. Viral infections can exacerbate symptoms of muscle weakness, and the clinical status of patients with MG may influence the outcomes of such infections. Here, we identified factors of symptom exacerbation, severe SARS-CoV-2 infection, and pneumonia in patients with MG who are infected with SARS-CoV-2.

**Methods:**

The clinical characteristics and outcomes of 341 MG patients infected with SARS-CoV-2 across multiple regions in China were determined.

**Results:**

The median age of the patients was 49 years (range: 35–60 years) and the median disease duration was 4 years (range: 2–8 years). Among the patients, 67 (49.0%) were male and 174 (51.0%) were female. Multivariate analysis indicated that thymectomy [OR, 1.654 (95% CI, 1.036–2.643); *p* = 0.035], severe SARS-CoV-2 infection [OR, 4.275 (95% CI, 2.206–8.286); *p* < 0.001], and pyridostigmine bromide [OR, 1.955 (95% CI, 1.192–3.206); *p* = 0.008] were associated with exacerbation of MG symptoms in patients infected with SARS-CoV-2. Age was significantly associated with severe SARS-CoV-2 infection [OR, 1.023 (95% CI, 1.001–1.046); *p* = 0.008], while patients with cardiac/vascular comorbidities exhibited an increased likelihood of severe SARS-CoV-2 infection [OR, 3.276 (95% CI, 1.027–10.449); *p* = 0.045]. Likewise, steroid treatment [OR, 6.140 (95% CI, 2.335–16.140); *p* < 0.001] was associated with a significantly increased likelihood of severe SARS-CoV-2 infection compared with symptomatic treatment. Additionally, gender [OR, 0.323 (95% CI, 0.120–0.868); *p* = 0.025] and SARS-CoV-2 severity [OR, 6.067 (95% CI, 1.953–18.850); *p* = 0.002] were associated with the occurrence of pneumonia.

**Conclusion:**

We identified factors that were associated with the exacerbation of MG symptoms in patients infected with SARS-CoV-2, including thymectomy, severe SARS-CoV-2 infection, and the use of pyridostigmine bromide. Due to the retrospective nature of the study, these findings should be interpreted as associations rather than predictive factors. However, the results confirm the established relationships between severe SARS-CoV-2 infection and age, cardiovascular comorbidities, and the use of steroid treatment, suggesting that these factors should be considered when managing MG patients during SARS-CoV-2 infection.

## 1 Introduction

Myasthenia gravis (MG) is an autoimmune disease that primarily affects the neuromuscular junction, leading to deterioration of the skeletal muscles. Research suggests that the symptomology is mediated by acetylcholine receptor (AChR) antibodies, cellular immunity, and the complement system, with primary involvement of the postsynaptic AChR (1–3). The primary treatment for MG is immunomodulation, commonly involving steroids, cyclophosphamide, azathioprine, eculizumab, and rituximab (4). However, patients undergoing these treatments face an elevated risk of infection. Furthermore, due to immunotherapy-induced immunosuppression and potential respiratory and bulbar muscle weakness, MG patients may be at higher risk for adverse outcomes compared to the general population. In particular, viral infections pose a significant risk factor for disease exacerbation (5, 6).

A correlation exists between viral infections and the development of autoimmune diseases (7). In particular, the host-dependent characteristics of viruses, which are more prone to gene integration and antigen modification than prokaryotic microorganisms, increase the occurrence of immune responses to novel autoantigens (8). MG has been associated with several viral infections, including Epstein–Barr virus, hepatitis E virus, West Nile virus, and human parvovirus B19 (9–12). A close interaction exists between SARS-CoV-2 and the individual’s immune system (13). Specifically, SARS-CoV-2 infection can induce a range of symptoms by affecting the respiratory, cardiac, immune, digestive, urinary, and neurological systems, potentially exacerbating MG or triggering a MG crisis (14).

Current research on MG patients infected with SARS-CoV-2 primarily focuses on case reports and descriptive studies. Japanese researcher Masahiro Mimori reported a case involving a patient who continued eculizumab treatment for MG after SARS-CoV-2 infection, which led to a favorable outcome (15). Neurologists from the United States (5) and Brazil (16) have reported descriptive statistics from small cohorts of MG patients infected with SARS-CoV-2. A study conducted in India demonstrated that SARS-CoV-2 infection was associated with anxiety, depression, and sleep quality in MG patients (17). In addition, the American researcher Lakshmi Prasanna Digala found that MG patients who tested positive for SARS-CoV-2 experienced a prolonged average hospital stay (18).

MG is a rare disease; however, China’s large population base results in a relatively high number of MG cases. Currently, there is limited research into the changes in MG symptoms following SARS-CoV-2 infection among the Chinese population. Therefore, we proposed a retrospective approach to investigate SARS-CoV-2–infected MG patients in China from January 2023 to December 2023 to identify predictive factors for MG symptom exacerbation, severe SARS-CoV-2 infection, and COVID-19 pneumonitis. The objective was to establish a foundation for medical personnel to assess the condition of MG patients with SARS-CoV-2 infection.

## 2 Methods

### 2.1 Study design and patient selection

From January to December 2023, all patients meeting the diagnostic criteria for MG were followed at the Myasthenia Gravis Treatment Center in Hebei Province. The diagnosis was based on typical clinical features of MG (characterized by fluctuating muscle weakness) and required meeting at least one of the following three criteria: pharmacological testing, electrophysiological characteristics, or positive serological testing for antibodies such as AChR or muscle-specific kinase antibodies (19).

All patients at the Myasthenia Gravis Treatment Center were informed of the study via SMS. Patient with positive SARS-CoV-2 nucleic acid and/or antigen tests were included. Infection was confirmed by positive throat or nasal swabs via real-time polymerase chain reaction testing for SARS-CoV-2 RNA and/or by detecting viral antigens in respiratory specimens with colloidal gold and immunofluorescence methods. Pre-infection data were collected from each patient’s medical records, and the infection course was assessed through telephone consultations with patients or their attending physicians. Informed consent was obtained from all participants in accordance with the procedures approved by the Ethics Committee of Shijiazhuang People’s Hospital.

### 2.2 Clinical data and evaluations

Clinical information was collected, including: patient source, age, gender, disease duration, and the maximum severity clinical classification of the Myasthenia Gravis Foundation of America (MGFA); history of cancer, cardiovascular/cerebrovascular diseases, hypothyroidism, hyperthyroidism, hypertension, and diabetes; vaccination status, methods of SARS-CoV-2 diagnosis, and temperature type; surgeries such as thymectomy or current thymoma; AChR antibody status, and medication history.

Exacerbation of MG was assessed by deterioration in one category of the MGFA classification or an increase of at least two points on the Activities of Daily Living scale (20). The severity of SARS-CoV-2 infection was classified on a 6-point scale: (1 = asymptomatic SARS-CoV-2; 2 = isolated symptoms, such as loss of smell or headache; 3 = mild infection, e.g., fatigue, cold, or cough; 4 = flu-like illness not requiring hospitalization; 5 = hospitalized patients requiring oxygen therapy; 6 = severe SARS-CoV-2 pneumonia). Severe SARS-CoV-2 infection was defined as a score of ≥5. Clinicians diagnose SARS-CoV-2 pneumonia based on examination findings and clinical assessment.

### 2.3 Statistical analysis

Numerical data are presented as the mean ± standard deviation or the median (interquartile range) to provide information on central tendency and dispersion. Categorical data are reported as absolute numbers and percentages to clearly demonstrate comparisons between groups. Univariate logistic regression analysis was conducted to identify potential factors associated with the primary outcome. Variables were selected based on clinical experience and prior studies, using a *p*-value <0.30 as the selection criterion to ensure the inclusion of potential confounders in the multivariate analysis. Subsequently, stepwise regression was employed for multivariate logistic regression analysis to identify independent predictors and the odds ratio (OR) and 95% confidence interval (CI) determined for each. All statistical analyses were performed using SPSS version 22.0, which provides robust regression analysis capabilities. Statistical significance was assessed using two-tailed tests, with a *p*-value <0.05 considered statistically significant.

## 3 Results

### 3.1 Geographic distribution

**Figure 1:**
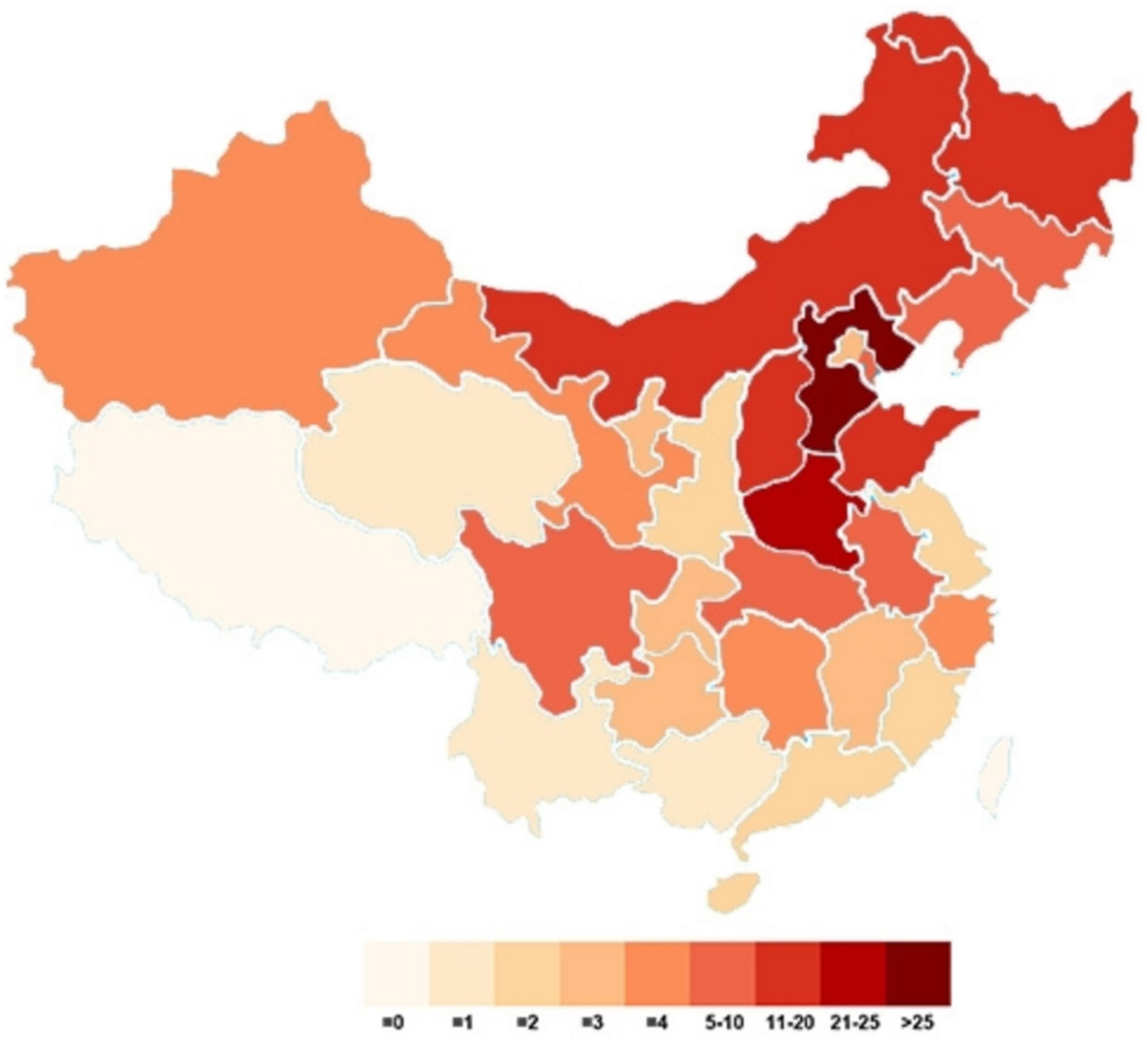
Geographical distribution of the study population.

This study included a total of 341 patients, with samples sourced from 28 provinces, 4 autonomous regions, and 4 municipalities in China. Among the patients, 182 (53.38%) were from Hebei and Henan, accounting for more than half of the study cohort. The next highest numbers of participants were from Heilongjiang, Inner Mongolia, Shandong, and Shanxi, with participant counts ranging from 10 to 20. The remaining participants were from various locations. Figure 1 illustrates the distribution of the study participants.

### 3.2 Descriptive characteristics

The study involved 341 patients diagnosed with MG who were infected with SARS-CoV-2. The clinical characteristics of these patients are presented in Tables 1, 2. The median age of the patients was 49 years (range: 35–60 years), while the median duration of the disease was 4 years (range: 2–8 years). Among the patients, 167 (49.0%) were male and 174 (51.0%) were female, yielding a male-to-female ratio of 167:174. Patients classified as MGFA II comprised the largest proportion of the MGFA classification, totaling 119 (34.9%). A total of 167 patients presented with comorbidities, including 14 cases (4.1%) of cancer, 19 cases (5.6%) of cardiac/vascular disease, 6 cases (1.8%) of hypothyroidism, 11 cases (3.2%) of hyperthyroidism, 76 cases (22.3%) of hypertension, and 41 cases (12%) of diabetes mellitus. Among the patients, 176 (51.6%) had received vaccination against SARS-CoV-2. A total of 276 patients (80.9%) reported experiencing fever, with a median duration of 2 days (range: 1–3 days). Following SARS-CoV-2 infection, the proportion of MG patients exhibiting worsening symptoms increased from 4.4% prior to infection to 5.6%. A total of 157 patients (65.1%) underwent treatment, including 26 (7.6%) receiving steroid therapy, 35 (10.3%) a combination of steroids and immunosuppressive agents, and 96 (28.1%) immunosuppressive agents.

**Table 1:** Overall, descriptive characteristics of the 341 patients suffering from myasthenia gravis and SARS-CoV-2 infection.

Parameter	*N*	%	Parameter	*N*	%
Gender			Medium fever	54	15.8
Male	167	49.0	High fever	151	44.3
Female	174	51.0	Thymectomy	190	55.9
MGFA classification before infection			Thymoma	95	50
I	111	32.6	Symptoms (before SARS-CoV-2 infection)		
II	115	33.7	Ptosis	87	25.5
III	55	16.1	Bulbar weakness	18	5.3
IV-V	60	17.6	Limb weakness	15	4.4
MGFA Classification during infection			Ptosis and Bulbar weakness	23	6.7
I	106	31.1	Ptosis and limb weakness	10	2.9
II	119	34.9	Bulbar and limb weakness	11	3.2
III	44	12.9	Ptosis, Bulbar and limb weakness	27	7.9
IV-V	72	21.1	Symptoms (after SARS-CoV-2 infection)	135	39.6
Comorbidity	167	49.0	Ptosis	35	10.3
Cancer	14	4.1	Bulbar weakness	13	3.8
Cardiac/vascular comorbidity	19	5.6	Limb weakness	19	5.6
Hypothyroidism	6	1.8	Ptosis and Bulbar weakness	18	5.3
Hyperthyroidism	11	3.2	Ptosis and limb weakness	7	2.1
hypertension	76	22.3	Bulbar and limb weakness	16	4.7
Diabetes	41	12.0	Ptosis, Bulbar and limb weakness	27	7.9
Vaccines			Treatment		
Not vaccinated	165	48.4	Symptomatic treatment	184	54
Partial vaccination	32	9.4	Steroid	26	7.6
Completion of vaccination	116	34.0	Steroids and immunosuppressive agents	35	10.3
Booster dose	28	8.2	Immunosuppressive agents	96	28.1
diagnostic modalities			Immune globulin	10	2.9
nucleic acid testing	119	34.9	Pyridostigmine bromide	113	33.1
Detection of antigens	174	51.0	Azathioprine, AZA	74	21.7
Nucleic acid and antigen Detection	48	14.1	Tacrolimus, FK560	45	13.2
Body temperature			Cyclosporin	9	2.7
Normal	65	19.1	Cyclophosphamide	7	2.1
Low fever	71	20.8	Pneumonia	50/94	53.2

**Table 2:** Overall, descriptive characteristics of the cohort of 341 patients suffering from myasthenia gravis and SARS-CoV-2 infection.

Parameter	Median	Q1	Q3
Age (year)	49	35	60
ADL scale before SARS-CoV-2 infection	2	1	4
ADL scale during SARS-CoV-2 infection	4	2	5
Disease duration (year)	4	2	8
Duration of fever (day)	2	1	3
Pyridostigmine bromide (mg)	180	120	180
AchR-Ab (nmol/L)	6.17	1.19	14.92

In the univariate analysis, MGFA classification (*p* = 0.041), thymectomy (*p* = 0.017), treatment (*p* = 0.016), severe SARS-CoV-2 (*p* < 0.001), and pyridostigmine bromide (*p* = 0.002) were significantly associated with the exacerbation of MG symptoms in patients infected with SARS-CoV-2. A binary logistic regression was conducted using the exacerbation of MG symptoms in patients infected with SARS-CoV-2 as the dependent variable and variables with *p* < 0.3 from the univariate analysis as independent variables. Patients who underwent thymectomy exhibited a higher likelihood of exacerbation of MG symptoms (OR, 1.654 [95% CI, 1.036–2.643]; *p* = 0.035), while the likelihood of exacerbation of MG symptoms in patients with severe SARS-CoV-2 infection was 4.275 times that of patients with mild SARS-CoV-2 infection [OR, 4.275 (95% CI, 2.206–8.286); *p* < 0.001]. The use of pyridostigmine bromide was associated with the exacerbation of MG symptoms in patients infected with SARS-CoV-2 [OR, 1.955 (95% CI, 1.192–3.206); *p* = 0.008], indicating a 1.955-fold increase in the risk of exacerbation of MG symptoms (Table 3).

**Table 3:** Univariate and multivariate logistic regression analysis of risk factors for aggravating symptoms in patients suffering from myasthenia gravis and SARS-CoV-2 infection.

Variables	Unvariable		Multivariable	
	OR (95%CI)	*P* value	OR (95%CI)	*P* value
Age	1.008 (0.995, 1.022)	0.227		
Gender (male)	1.286 (0.832, 1.987)	0.258		
Time from onset to 2023	0.987 (0.949, 1.026)	0.509		
MGFA classification		**0.041**		
I	1 [Reference]			
II	1.717 (0.993, 2.970)	0.053		
III	1.032 (0.485, 2.198)	0.934		
IV-V	2.212 (1.192, 4.106)	**0.012**		
Cancer	2.100 (0.714, 6.193)	0.179		
Cardiac/vascular comorbidity	0.691 (0.256, 1.863)	0.465		
Hypothyroidism	0.300 (0.035, 2.596)	0.274		
Hyperthyroidism	0.868 (0.249, 3.024)	0.824		
Hypertension	1.066 (0.634, 1.793)	0.808		
Diabetes	1.224 (0.633, 2.366)	0.548		
Thymectomy	1.721 (1.104, 2.685)	**0.017**	1.654 (1.036, 2.643)	**0.035**
Vaccines				
Not vaccinated	1 [Reference]			
Partial vaccination	1.435 (0.666, 3.093)	0.357		
Completion of vaccination	1.552 (0.955, 2.522)	0.076		
Booster dose	1.025 (0.444, 2.366)	0.954		
Immune globulin	0.372 (0.078, 1.780)	0.216	4.252 (0.798, 22.639)	0.095
Treatment		**0.016**		
Symptomatic treatment	1 [Reference]			
Steroid	2.808 (1.188, 6.635)	**0.019**		
Steroids and immunosuppressive agents	1.115 (0.536, 2.317)	0.771		
Immunosuppressive agents	0.643 (0.380, 1.089)	0.100		
Severe SARS-CoV-2	0.208 (0.109, 0.397)	**<0.001**	4.275 (2.206, 8.286)	**<0.001**
Body temperature		0.271		
Normal	1 [Reference]			
Low fever	1.987 (0.977, 4.041)	0.058		
Medium fever	1.664 (0.777, 3.566)	0.190		
High fever	1.687 (0.903, 3.151)	0.101		
Pyridostigmine bromide	2.068 (1.306, 3.275)	**0.002**	1.955 (1.192, 3.206)	**0.008**
AchR-Ab	1.010 (0.976–1.046)	0.568		

Bold values: *P* value < 0.05.

Furthermore, univariate analysis results indicated that age (*p* = 0.022), cardiac/vascular comorbidity (*p* = 0.050), immunosuppressive therapy (*p* < 0.001), and pyridostigmine bromide (*p* = 0.006) were associated with severe SARS-CoV-2 infection. A binary logistic regression was conducted using the severity of SARS-CoV-2 infection as the dependent variable and the variables with *p* < 0.3 from the univariate analysis as independent variables. The results of the multivariate analysis indicated that age was associated with severe SARS-CoV-2 infection [OR, 1.023 (95% CI, 1.001–1.046); *p* = 0.008] and also that, for each additional year of age, the likelihood of experiencing severe SARS-CoV-2 infection increased by 2.3%. Patients with cardiac/vascular comorbidities exhibited an increased likelihood of severe SARS-CoV-2 infection [OR, 3.276 (95% CI, 1.027–10.449); *p* = 0.045]. Steroid therapy [OR, 6.140 (95% CI, 2.335–16.140); *p* < 0.001] was associated with a significantly increased likelihood of severe SARS-CoV-2 infection compared to patients not receiving immunosuppressive therapy. However, neither the combination of steroids and immunosuppressive therapy [OR, 1.674 (95% CI, 0.589–4.761); *p* = 0.334] nor immunosuppressive therapy alone [OR, 0.920 (95% CI, 0.395–2.144); *p* = 0.847] demonstrated any significant effects compared to patients not receiving immunosuppressive therapy (Table 4).

**Table 4:** Univariate and multivariate logistic regression analysis of risk factors for severe SARS-CoV-2 infection in patients suffering from myasthenia gravis.

Variables	Unvariable		Multivariable	
	OR (95%CI)	*P* value	OR (95%CI)	*P* value
Age	1.022 (1.003, 1.042)	**0.022**	1.023 (1.001, 1.046)	**0.040**
Gender (male)	1.823 (0.991, 3.354)	0.054		
Time from onset to 2023	0.989 (0.937, 1.044)	0.693		
MGFA Classification		0.116		
I	1 [Reference]			
II	1.130 (0.503, 2.536)	0.767		
III	1.741 (0.658, 4.608)	0.264		
IV-V	2.421 (1.077, 5.445)	**0.032**		
Cancer	1.547 (0.417, 5.747)	0.514		
Cardiac/vascular comorbidity	2.769 (1.002, 7.652)	**0.050**	3.276 (1.027, 10.449)	**0.045**
Hypothyroidism	1.114 (0.127, 9.731)	0.922		
Hyperthyroidism	0.868 (0.249, 3.024)	0.824		
Hypertension	0.694 (0.322, 1.496)	0.351		
Diabetes	0.947 (0.377, 2.378)	0.907		
Thymectomy	1.327 (0.725, 2.429)	0.360		
vaccines				
Not vaccinated	1 [Reference]			
Partial vaccination	1.497 (0.587, 3.820)	0.399		
Completion of vaccination	0.794 (0.400, 1.575)	0.509		
Booster dose	0.891 (0.285, 2.781)	0.843		
Treatment		**<0.001**		
Symptomatic treatment	1 [Reference]			
Steroid	5.714 (2.365, 13.809)	0.000	6.140 (2.335, 16.140)	**<0.001**
Steroids and immunosuppressive agents	1.379 (0.519, 3.668)	0.519	1.674 (0.589, 4.761)	0.334
Immunosuppressive agents	0.775 (0.354, 1.696)	0.524	0.920 (0.395, 2.144)	0.847
Immune globulin	1.639 (0.203, 13.219)	0.643		
Pyridostigmine bromide	2.322 (1.276, 4.226)	**0.006**		
Body temperature		0.888		
Normal	1 [Reference]			
Low fever	1.449 (0.552, 3.807)	0.452		
Medium fever	1.239 (0.432, 3.556)	0.690		
High fever	1.346 (0.570, 3.179)	0.497		

Bold values: *P* value < 0.05.

Additionally, univariate analysis results indicated that thymectomy (*p* = 0.048), exacerbation of MG symptoms (*p* = 0.024), and severe SARS-CoV-2 infection (*p* = 0.007) were associated with the occurrence of pneumonia. A binary logistic regression was again conducted using the occurrence of pneumonia as the dependent variable and variables with *p* < 0.3 from the univariate analysis as independent variables. Gender [OR, 0.323 (95% CI, 0.120–0.868); *p* = 0.025] and SARS-CoV-2 severity [OR, 6.067 (95% CI, 1.953–18.850); *p* = 0.002] were identified as factors associated with the occurrence of pneumonia (Table 5).

**Table 5:** Univariate and multivariate logistic regression analysis of risk factors for COVID-19 pneumonia in patients suffering from myasthenia gravis and SARS-CoV-2 infection.

Variables	Unvariable		Multivariable	
	OR (95%CI)	*P* value	OR (95%CI)	*P* value
Age	1.010 (0.984, 1.036)	0.456		
Gender (male)	0.597 (0.264, 1.352)	0.216	0.323 (0.120, 0.868)	0.025
Time from onset to 2023	0.991 (0.881, 1.114)	0.882		
MGFA classification		0.191		
I	1 [Reference]			
II	1.915 (0.630, 5.822)	0.252		
III	1.167 (0.303, 4.499)	0.823		
IV-V	3.284 (1.036, 10.406)	**0.043**		
Cancer	0.878 (0.053, 14.458)	0.927		
Cardiac/vascular comorbidity	1.864 (0.437, 7.943)	0.400		
Hypothyroidism	0.878 (0.053, 14.458)	0.927		
Hyperthyroidism	0.875 (0.118, 6.486)	0.896		
Hypertension	0.667 (0.255, 1.740)	0.407		
Diabetes	0.721 (0.223, 2.334)	0.585		
Thymectomy	2.327 (1.006, 5.383)	**0.048**	2.245 (0.911, 5.531)	0.079
Vaccines		0.481		
Not vaccinated	1 [Reference]			
Partial vaccination	1.131 (0.241, 5.304)	0.876		
Completion of vaccination	0.519 (0.205, 1.312)	0.166		
Booster dose	0.543 (0.129, 2.287)	0.405		
Immunosuppressive therapy		0.391		
Symptomatic treatment	1 [Reference]			
Steroid	2.130 (0.589, 7.706)	0.249		
Steroids and Immunosuppressive agents	0.568 (0.143, 2.262)	0.422		
Immunosuppressive agents	0.697 (0.246, 1.975)	0.497		
Exacerbation of MG	2.625 (1.139, 6.051)	**0.024**		
Severe SARS-CoV-2 infection	3.828 (1.431, 10.238)	**0.007**	6.067 (1.953, 18.850)	0.002
Body temperature		0.569		
Normal	1 [Reference]			
Low fever	1.222 (0.341, 4.381)	0.758		
Medium fever	2.667 (0.529, 13.433)	0.234		
High fever	0.957 (0.321, 2.854)	0.936		
Immune globulin	1.792 (0.157, 20.463)	0.639		

Bold values: *P* value < 0.05.

## 4 Discussion

MG has been found to be associated with various viral infections, including Epstein–Barr virus hepatitis E virus, West Nile virus, and human parvovirus B19 (9–12). A study reported a case of an Egyptian male patient who developed typical MG symptoms during the course of chronic hepatitis C complicated by cirrhosis (21). The hepatitis C pathogenic mechanism is known to be similar to that of herpes simplex virus and HIV (22) and hepatitis C may induce MG through a cross-reactive mechanism between viral epitopes and AChRs. Research has also reported new-onset MG patients following SARS-CoV-2 infection, potentially increasing the risk of MG through a molecular mimicry mechanism, wherein viral proteins are similar to components of the AChR, possibly triggering an autoimmune response (23).

The current study identified factors that were associated with the exacerbation of MG symptoms in patients with SARS-CoV-2, including thymectomy, severe SARS-CoV-2 infection, and the use of pyridostigmine bromide. Due to the retrospective nature of the study, these findings should be interpreted as associations rather than predictive factors. The results confirm the established relationships between severe SARS-CoV-2 infection and age, cardiovascular comorbidities, and the use of steroid treatment, suggesting that these factors should be considered when managing MG patients during SARS-CoV-2 infection.

The median age of the study population was 49 years, with a median disease duration of 4 years, which is consistent with the findings of Indian neurologist Jayantee Kalita (17). We observed that this median age is lower than that reported by Czech neurologist Michala Jakubíková (24), potentially due to differences in the definitions of severe SARS-CoV-2 between the two studies. It is important to emphasize that our study did not include MG patients who died due to SARS-CoV-2 infection. Previous research has shown that older patients generally have a poorer prognosis, which may explain the relatively younger age of our study population.

The thymus is a crucial organ involved in the development of T cell-mediated immunity and plays a key role in central immune regulation. Abnormalities in the thymus are closely associated with the AChR subtype in MG (25). Retrospective studies suggest that thymectomy may bring potential benefits to non-thymomatous MG patients; however, the rates of clinical improvement or remission vary significantly. Some observational studies have not demonstrated the benefits of thymectomy, which may be due to the effectiveness of current immunotherapies (26). For example, one study showed that clinical improvement was more evident in the first few years following thymectomy, but after 5 years, outcomes in patients undergoing surgery were similar to those managed with pharmacological treatment alone (27). Furthermore, a systematic review found that patients under 45 years of age are more likely to achieve complete stable remission during follow-up after thymectomy (28). Regarding immune function and infection risk, previous studies have suggested that reduced T cell diversity may make individuals more susceptible to viral infections (29).

In our study, 92 thymoma patients underwent thymectomy. We found that thymectomy was associated with exacerbation of MG symptoms, which may be linked to reduced T cell diversity and diminished immune regulatory capacity after surgery. Further analysis of the time elapsed since thymectomy could help determine if this factor influences infection risk. If thymectomy was performed for thymoma treatment, patients may have a more complex immune profile, potentially making them more vulnerable to viral infections. Additionally, some patients in our study continued to use steroids or other immunosuppressants following thymectomy, which could further suppress immune function and increase infection risk. Future studies should explore the relationships between thymectomy background (such as thymomatous vs. non-thymomatous cases), concurrent medication use, and infection risk in greater detail to guide tailored treatment strategies for MG patients, especially those who have undergone thymectomy and are at risk of infections.

SARS-CoV-2 infection is associated with pneumonia and myositis, which may exacerbate MG (30), consistent with the findings of our study. Interestingly, pyridostigmine bromide was associated with infection-related exacerbation of MG symptoms, which may be related to the exacerbation of swallowing and respiratory difficulties in patients with bulbar MG. However, pyridostigmine bromide is also often used as monotherapy in patients with milder MG symptoms, such as ocular or limb MG, suggesting there may be additional contributing factors. For instance, during an infection, the increased neuromuscular transmission load and potential drug interactions might further exacerbate overall symptoms. Additionally, in clinical practice, more severe MG cases are commonly managed with corticosteroids or immunomodulators in combination therapy, whose immunomodulatory effects under infectious conditions warrant further investigation.

The results confirm the established relationships between severe SARS-CoV-2 infection and age, comorbid cardiovascular diseases, and steroid treatment. Previous studies indicated that older SARS-CoV-2 patients had a higher likelihood of experiencing severe infection (31), which was consistent with our findings. Comorbidities such as hypertension, diabetes, cardiovascular diseases, and respiratory diseases could represent significant risk factors for complications in patients with aggravated SARS-CoV-2 infections (32, 33). Our results showed that cardiovascular diseases were significantly related to severe SARS-CoV-2 infection, while the associations whereas hypertension and diabetes were not. This result could be attributed to the fact that our study population consisted of MG patients infected with SARS-CoV-2, who had a relatively younger median age. Steroids are considered effective in reducing mortality rates among patients with SARS-CoV-2 pneumonia, but their impact on improving clinical outcomes and reducing mortality remained uncertain (34). Our findings indicated that steroid treatment was associated with severe SARS-CoV-2 infection, which might have been related to the increased risk of infection associated with steroid use. Indeed, steroid treatment might not benefit all SARS-CoV-2 patients (35). Studies have shown that immunosuppressed patients have a higher risk of more severe SARS-CoV-2 disease progression. However, there is growing evidence suggesting that immunosuppression might play a protective role by reducing immune responses that lead to cytokine storms and clinical deterioration (36, 37). Nonetheless, in the current study, the results for both monotherapy with immunosuppressants and combination therapy with immunosuppressants and steroids were not significant.

## 5 Limitations

First, this study is a retrospective study, and the results primarily reveal associations among various factors but do not sufficiently demonstrate causal relationships. Additionally, this study did not compare other viral agents. Future research will need to consider employing a prospective cohort study design to more effectively assess causal relationships, while also further collecting data on specific pathogenic microorganisms during exacerbations in MG patients for comparison. Second, the case data was collected from specific provinces or regions, which may introduce geographic distribution bias. Therefore, future research is recommended to collect data in a more geographically balanced manner or include cases from more regions to enhance the representativeness and generalizability of the results.

## 6 Conclusion

We identified factors associated with the exacerbation of MG in patients infected with SARS-CoV-2, including thymectomy, severe SARS-CoV-2 infection, and the use of pyridostigmine bromide. Due to the retrospective nature of the study, these findings should be interpreted as associations rather than predictive factors.

## Data Availability

The original contributions presented in the study are included in the article/supplementary material, further inquiries can be directed to the corresponding author/s.
